# Influence of Mineral Additives on Strength Properties of Standard Mortar

**DOI:** 10.3390/ma17164158

**Published:** 2024-08-22

**Authors:** Grzegorz Rogojsz, Tomasz Rudnicki

**Affiliations:** Faculty of Civil Engineering and Geodesy, Military University of Technology, 2 Gen. Sylwestra Kaliskiego Str., 00-908 Warsaw, Poland; grzegorz.rogojsz@wat.edu.pl

**Keywords:** mineral additives, compressive and bending strength, standard mortar

## Abstract

In the article, the authors presented the results of research on the assessment of the effect of selected mineral additives on the strength properties of the standard mortar. The modification of the composition of the standard mortar made on the basis of CEM I 42.5R cement and quartz sand consisted of using seven selected mineral additives in the form of compacted microsilica, Mikrosill microsilica, limestone flour, glass flour, glass granulate, basalt flour, and fly ash in the amounts of 10 and 20% in relation to cement as its substitute. Reducing the share of cement in the standard mortar by 10% has a beneficial effect on improving the compressive strength by over 40% with the addition of microsilica, and in the case of bending strength, even by 10%.

## 1. Introduction

Due to the rapidly developing economy, the numerous resources of building materials used so far are being used very quickly. Therefore, it is very important to use a number of mineral additives and chemical admixtures for concrete [1,2,3,4], thanks to which it is possible to optimize the composition of the concrete mix in terms of its beneficial impact on the environment, which was discussed in [5]. At the stage of optimizing the composition of a concrete mix with a reduced negative impact on the environment, it is important to reduce the share of cement in the concrete mix. Such a procedure will allow for a significant reduction in CO_2_ emissions to the atmosphere, which, with a cement consumption of 4 gigatons per year [6], will significantly affect the environment, assuming traditional cement production as the main binding material in concrete [7,8]. Since the production of Portland cement is characterized by the highest energy consumption [9], which causes CO_2_ emissions in the amount of about 0.9 tons per 1 ton of cement produced [10], according to [11], this constitutes about 5% of the total anthropogenic CO_2_ emissions in the world. In order to reduce CO_2_ emissions into the atmosphere resulting from the increased development of construction, an attempt was made to replace part of the cement with mineral additives. The influence of mineral additives in the form of zeolite and silica fume was discussed, among others, in [12,13,14,15]. The use of the metakaolinite additive was discussed in more detail in [16,17,18,19]. The authors of [20,21,22,23] presented the possibilities of using fly ash in mortars and cement concretes. In [24,25], the beneficial influence of quartz flour on the strength of cement concrete was presented. According to the results published in [26,27,28,29,30], the use of mineral additives is beneficial both in terms of environmental protection and contributes to increasing the strength parameters of mortars and cement concretes as well as improving the workability of the concrete mix [31,32,33]. In addition, mineral additives in the form of fly ash and silica fume are used in the production of UHPC concretes, as presented by the authors of [34,35]. Mineral additives in the form of silica fume, blast furnace ash, and slag are an important element in optimizing the composition of mixtures with high efficiency and compressive strength because they cause the cement matrix to seal, which significantly improves the properties of cement concrete. Analysis of the literature in this area prompted the authors to conduct research on the possibility of using mineral additives for cement mortars, which is the first stage of research, the results of which are presented in this article. The next stage is the introduction of selected additives as a component of the concrete mix in the scope of not only strength tests but also determining the durability of concrete. The main objective of the work is to verify the strength properties of cement mortars using various mineral additives, which are the main components of cement concrete. After obtaining positive results, the authors plan to perform tests on the designed concrete compositions for selected additives in the next stage. The new approach in the work is a wide spectrum of mineral additives subjected to tests and the search for the dependence of strength properties on the grain size and specific surface of mineral additives.

## 2. Materials and Methods

### 2.1. Materials

In order to assess the effect of individual mineral additives on the strength parameters of the standard mortar, the tests used standard sand from KWARCMIX (Smardzewice, Poland), which meets the requirements specified in the [36], and cement class CEM I 42.5R (Holcim Warsaw, Poland), hereinafter referred to as CEM I, with the parameters presented in Table 1.

**Figure 1:**
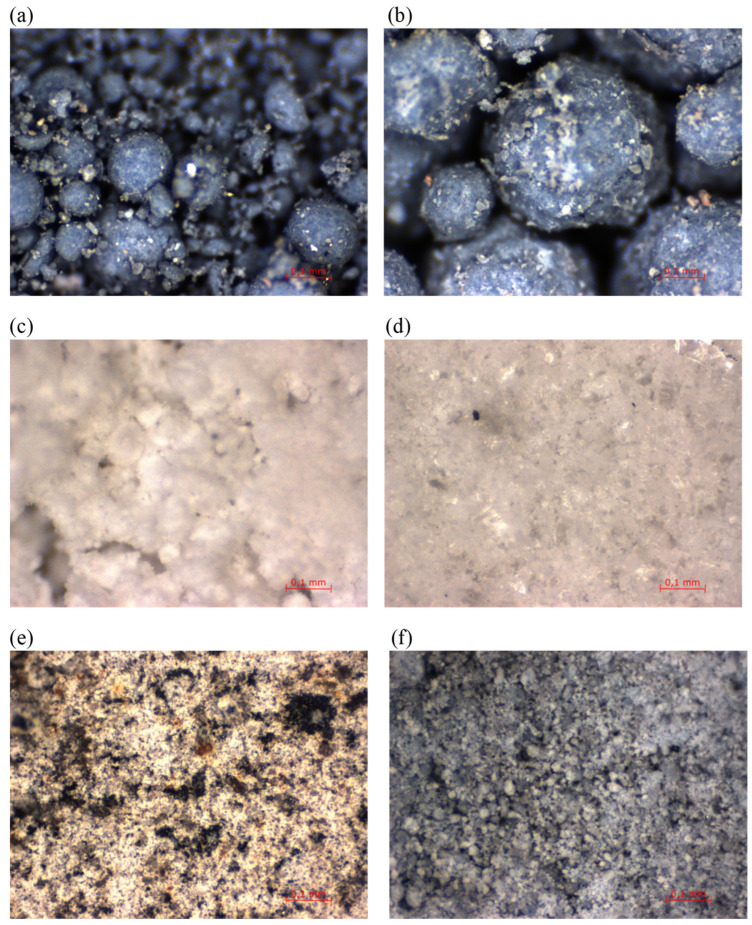
Mineral additives: (**a**) compacted microsilica; (**b**) Mikrosill+ microsilica; (**c**) limestone flour; (**d**) glass flour; (**e**) basalt dust; and (**f**) fly ash.

The mineral additives used were compacted microsilica (MZ) and Mikrosill+ (MK+) from Mikrosilika Trade, in the form of silica dust generated in arc furnaces during the production of metallic silicon and ferrosilicon alloys. Another additive is limestone flour (MW), used as a filler for mineral-asphalt mixtures, created by drying and grinding limestone, the main component of which is calcium carbonate. The influence of glass waste generated on the basis of construction glass with a hardness of 6–7 on the Mosh scale in the form of glass flour (MS) and glass granulate (GS) was also examined. Another additive analyzed is basalt dust (PB), also called basalt flour, which is waste generated during the processing of aggregate for the production of mineral-asphalt mines. The last additive is fly ash (FA), which is a fine-grained dust consisting of spherical, vitrified grains formed during the combustion of coal dust, mainly during the combustion of coal in power plants. The chemical composition of the individual additives is presented in Table 2, and the additives are shown in Figure 1.

**Figure 2:**
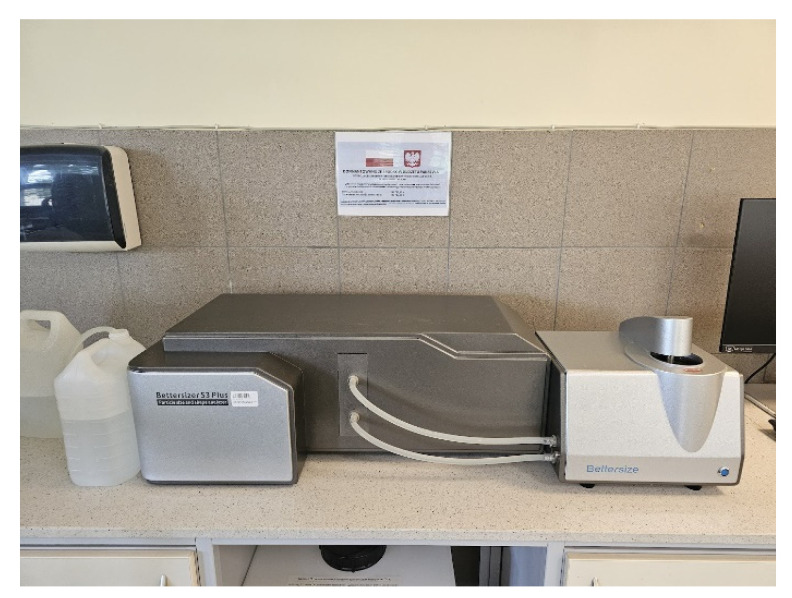
Bettersizer S3 plus Laser Particle Size measuring device.

**Figure 3:**
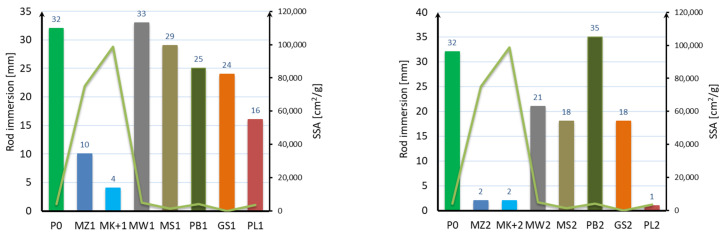
Consistency measurement results.

**Figure 4:**
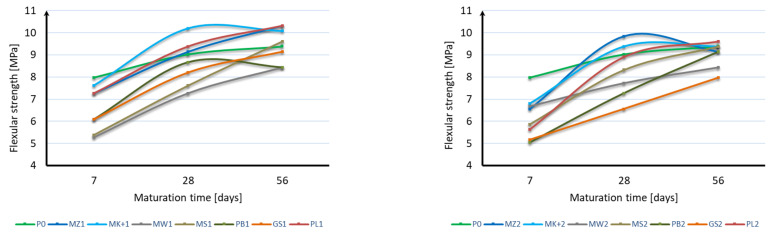
Flexural strength over time.

**Figure 5:**
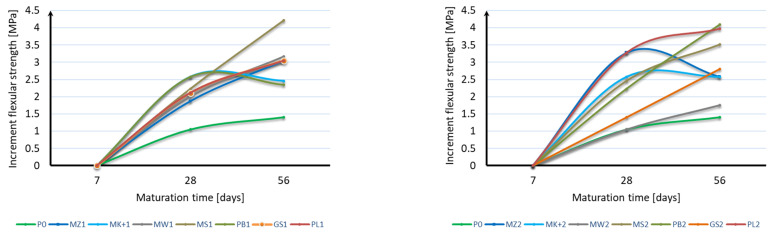
Increase in flexural strength over time.

Compacted microsilica and Mikrosill+ are characterized by a homogeneous structure in the form of spherical grains with a maximum size below 0.003 mm. Microsilica in loose clusters of grains of 0.1–0.2 mm in size, visible in Figure 1 and Figure 2, is created by the agglomeration of small particles, which immediately disintegrate after contact with water. Limestone flour consists of non-homogeneous grains, mainly below 0.06 mm in size, that do not combine with each other, creating a loose material in the form of dust, as shown in Figure 3. In contrast to limestone flour, glass flour is characterized by a very homogeneous structure, with grains below 0.06 mm in size (Figure 4). Basalt dust, such as limestone flour, is formed by non-homogeneous grains of crushed aggregate with a size below 0.08 mm (Figure 5). Fly ash is composed of spherical grains with a size of less than 0.02 mm, which tend to “stick together” and form larger clusters with a size of about 0.01 mm, as shown in Figure 1f.

### 2.2. Research Methods

Samples for laboratory tests were prepared in accordance with the procedure described in the PN-EN 196-1 standard, in the form of bars with dimensions of 40 mm × 40 mm × 160 mm. After mixing the materials in a standard mixer, the samples were compacted in two layers in three-part molds on a shaking table with 60 blows for each layer. Nine samples were prepared for each additive and three samples for each maturation time, i.e., for 7, 28, and 56 days of maturation of the mortar samples. In the first stage of the tests, 10% of the cement content was replaced with a mineral additive, and in the second stage, 20% of the cement content was replaced with a mineral additive. In order to compare the effect of individual additives and their content, a reference mortar without mineral additives was prepared, consisting of 450 g of cement, 1350 g of standard sand, and 225 g of water. The analysis of materials began with the determination of the grain size analysis of mineral additives and cement, together with the determination of the specific surface area. The measurement of particle size and specific surface area was performed using the Bettersizer S3 Plus Laser Particle Size Analyzer from Dandong Bettersize Instruments Ltd. (Dandong, China), as shown in Figure 2. The tests were carried out using distilled water as a medium for dispersing the measuring material. Since the mass of the sample used during the measurement is only 10 mg, it was decided to perform three tests for each mineral additive.

This study of the basic features of the designed mortars with mineral additives began with determining the consistency for each batch in the Vicat apparatus, equipped with a measuring pin with a diameter of 10.0 ± 0.05 mm and a measuring container with an internal diameter of 75 ± 10 mm and a height of 40.0 ± 0.2 mm. In order to determine the effect of mineral additives on the consistency of the mortar, it was decided to test the consistency in accordance with [37]. This study of the consistency in the Vicat apparatus made it possible to determine the effect of individual additives on the consistency of the mortar more precisely than the classical method using a flow table. This study of the strength features of the prepared mortars began with determining the bending and compression strengths of the designed mortar samples. This test was performed on previously prepared beams with dimensions of 40 mm × 40 mm × 160 mm, and a compressive strength test was performed on the beam halves remaining after the bending strength test. The bending strength and compressive strength tests were performed in accordance with the procedure specified in PN-EN 196-1 Cement Testing methods—part 1: Marking of Strength. Both tests were performed after 7, 28, and 56 days of curing the mortar samples.

After preparing the laboratory samples, strength tests were carried out. The tests began with determining the bending strength of 40 mm × 40 mm × 160 mm beams. The bending strength test was carried out in accordance with the procedure specified in PN-EN 196-1 Cement Testing methods—part 1: Strength Determination. A universal strength press with a measuring range of 0–50 kN, manufactured by Zakład Aparatury Naukowej in Krakow, was used to carry out the test. After performing the bending strength test on the halves of the beams remaining after the test, a compressive strength test was carried out. This test, such as the previous one, was carried out in accordance with the procedure specified in PN-EN 196-1 Cement Testing methods—part 1: Strength Determination. The test was carried out using a 5000 testing machine, manufactured by Testing Bluhm & Feuerherdt GmbH in Berlin, Germany, which allows for carrying out strength tests in the range of 0–250 kN and 0–5000 kN.

The description of the test sample symbols used is presented in Table 3.

## 3. Results

### 3.1. Analysis of Grain Size of Mineral Additives

In order to determine the effect of individual mineral additives on the strength properties of mortars, their grain size and specific surface area were tested. Since the analysis of the grain composition divided into specific fractions would be very complicated, it was decided to determine the grain size level of each additive using the D50 and D90 indexes. The D50 index means the maximum grain size expressed in μm, below which 50% of the material is contained, and the D90 index means the maximum grain size expressed in μm, below which 90% of the material is contained. The values of the D50, D90, and specific surface areas are listed in Table 3. The grain size and specific surface area tests of compacted microsilica and Mikrosill+ microsilica were performed after the additive had been stored in water for 10 days in order to break up the lumps of material (Table 4).

This research shows that microsilica has the largest specific surface area. Compared to cement, the surface area of compacted microsilica is almost 18 times larger, and that of Mikrosill+ microsilica is over 23 times larger. Limestone flour and basalt dust have a specific surface area slightly larger than cement, while fly ash has a slightly smaller surface area. Glass flour has a specific surface area three times smaller than cement, and glass granulate is 32 times smaller. The specific surface area results from the size of the material particles. It should therefore be stated that materials that are characterized by finer particles than cement have a larger specific surface area. This research confirms this thesis only in the case of microsilica, glass flour, and glass granulate. Fly ash, 90% of whose grains have a diameter smaller than 90% of cement grains, has a specific surface area smaller than cement. On the other hand, basalt dust and glass flour, 90% of whose grains have a diameter greater than 90% of cement grains, are characterized by a specific surface area greater than cement. It should therefore be stated that the size of the specific surface area, in addition to the size of the grain, is influenced by its shape. The more angular the grain, e.g., as a result of crushing, the greater its specific surface area.

### 3.2. Marking the Consistency of Standard Mortar

The results of the measurement of the consistency of the mortar with 10 and 20% mineral additives as a cement substitute are shown in Figure 3.

Based on the obtained results, it can be stated that with the addition of 10% and 20% of microsilica and 20% of fly ash, there was a significant decrease in the consistency of the mortar from 32 mm for the mortar without additives to 10 mm for 10% of the content of compacted microsilica and 2% for the content of 20% of compacted microsilica, Mikrosill+, and fly ash. In the case of 10% of the content of limestone flour and glass flour and 20% of the content of basalt dust, the consistency of the mortar is similar to that of the mortar without additives. A slight decrease in the consistency of 21 mm to 25 mm occurred when using 10% basalt dust, glass granulate, and 20% lime flour. A consistency twice as low was obtained for samples with 10% fly ash and 20% of the content of glass flour and glass granulate. In general, it can be observed that with the increase in the content of mineral additives, the consistency of the mixture decreases, the exception being the 20% content of basalt dust, for which the consistency increased.

Based on the presented results, it can be stated that there is a dependence of mortar consistency on the specific surface only in the case of both microsilicas, which are characterized by a specific surface above 80,000 mc^2^/g. In the case of the remaining additives, such a dependence does not occur for either the specific surface or the grain size. Attention should be paid to the results of the glass granulate (GS) consistency test, which, despite the smallest specific surface and the largest grains, caused a decrease in consistency in relation to the standard mortar without additives.

### 3.3. Designation of Flexural Strength

The flexural strength of the mortar samples was tested after 7, 28, and 56 days of curing. The results of the flexural strength test are presented in Table 5.

Based on the analysis of the obtained results, it can be stated that it is not possible to unequivocally determine the effect of mineral additives and their content on the flexural strength of mortar samples. In the case of strength after 7 days of maturation, all mineral additives are characterized by a strength lower by 5% to 37% than mortar samples without additives. The most favorable of all are samples with a 10% addition of both Mikrosilika (MZ1 and MK+1) and ash (PL1). In the flexural strength test after 28 days of maturation, samples with the addition of 10% and 20% Mikrosilika (MZ1, MK+1, MZ2, MK+2) and with a 10% addition of fly ash (PL1) achieved a flexural strength greater than samples without mineral additives. In the case of the flexural strength test, after 56 days of maturation, half of the samples achieved flexural strength equal to or greater than samples without additives. The lowest strength was characteristic for samples with 10% content of mineral additives in the form of limestone flour (MW1), basalt dust (PB1), and glass granulate (GS1) and with 20% content of mineral additives in the form of compacted microsilica (MZ2), limestone flour (MW2), basalt dust (PB2), and glass granulate (GS2). Analyzing the obtained results, it can be stated that the flexural strength does not depend directly on the specific surface area of the mineral additive used. Based on the flexural strength results, an analysis of the increase in flexural strength during the maturation of the samples was made for each additive, depending on its content. Figure 4 shows the results of the sample strength over time, while Figure 5 shows the strength increases over time. For each sample, the reference value was the bending strength after 7 days of curing. The strength increases were presented as the strength increase after 28 days and after 56 days relative to the strength after 7 days for each additive separately.

Based on the presented results, it should be stated that the applied mineral additives had a positive effect on the increase in flexural strength over time. In the case of using a 10% additive instead of cement, the same increases were obtained in samples with the addition of Mikrosill+ microsilica and basalt dust. The second group of additives having a similar effect on increasing flexural strength are compacted microsilica, limestone flour, glass granulate, and fly ash. The greatest increase in strength is characterized by glass flour, which is four times greater than the samples of mortar without additives. Analyzing the results of using a 20% additive instead of cement, it should be stated that the results are much more distributed; however, all additives also showed a greater increase in strength than the blank samples. In most cases, the increase in strength can be considered linear.

### 3.4. Designation of Compressive Strength

**Figure 6:**
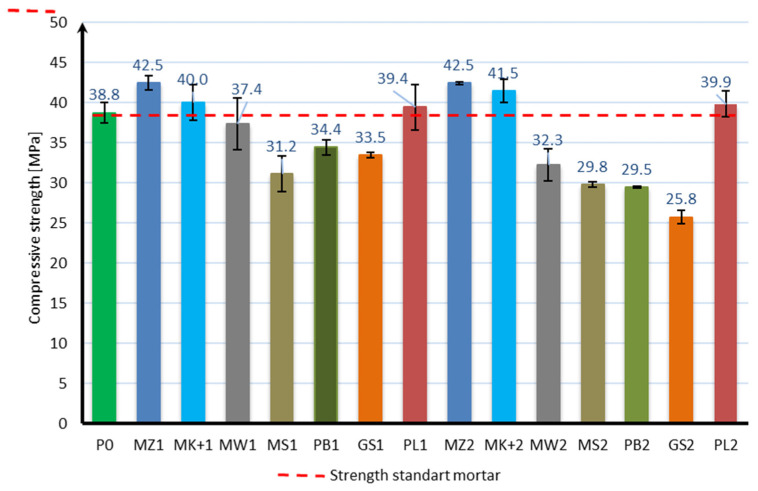
Compressive strength test results after 7 days.

**Figure 7:**
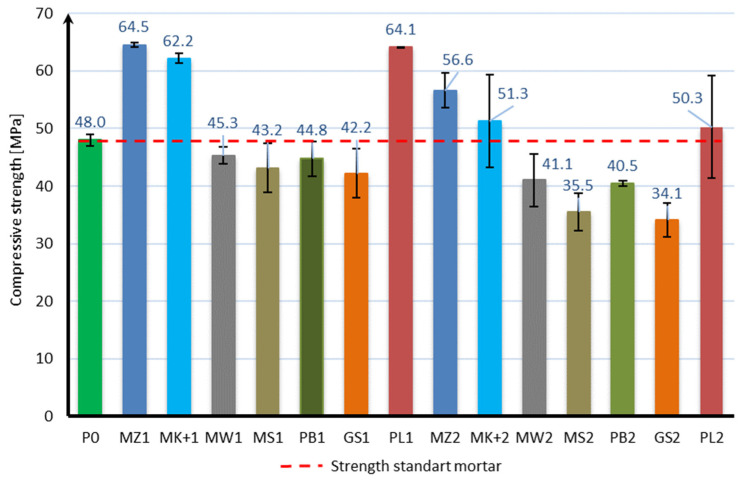
Compressive strength test results after 28 days.

**Figure 8:**
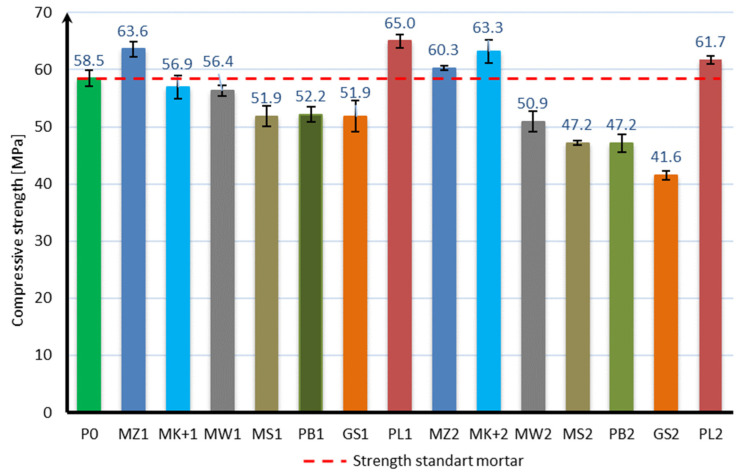
Compressive strength test results after 56 days.

**Figure 9:**
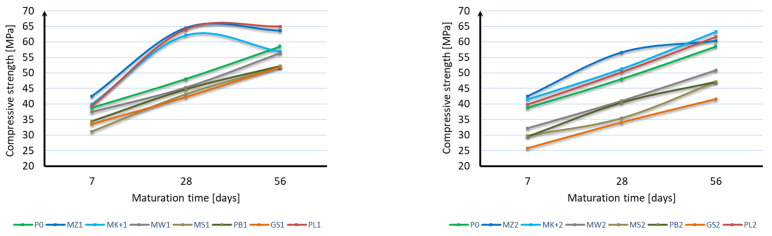
Compressive strength over time.

**Figure 10:**
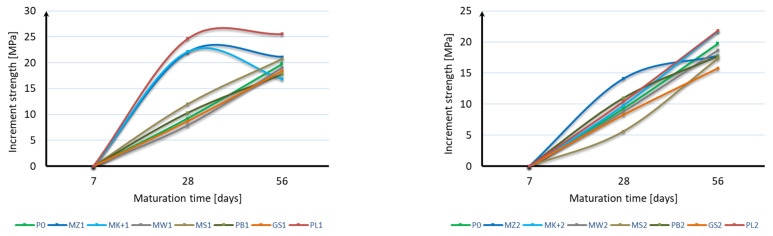
Increase in compressive strength over time.

The compressive strength of the mortar samples was performed in the same way as for the bending strength test after 7, 28, and 56 days of curing. The bending strength test results are shown in Figure 6, Figure 7 and Figure 8:

Based on the obtained results, it can be stated that the applied mineral additives do not significantly adversely affect the compressive strength of the samples. Already in the case of the compressive strength test after 7 days of curing, it can be seen that 6 samples are characterized by a strength greater than the samples of mortar without additives. These are samples with 10% content of additives in the form of microsilica (MZ1 and MK+1) and fly ash (PL1), as well as with 20% content of mineral additives in the form of microsilica (MZ2 and MK+2) and fly ash (PL2). The highest strength of 42.5 MPa was achieved by samples with the addition of 10% and 20% of compacted microsilica, which, in comparison to samples without additives, constitutes a 10% increase in compressive strength. In the case of the compressive strength test after 28 days of curing, the same samples are characterized by a strength greater than the samples without additives, as in the case of the test after 7 days. However, there was a significant increase in the strength of samples with the addition of microsilica and fly ash compared to samples without additives. Samples with a 10% addition of compacted microsilica, Mikrosill+ microsilica, and fly ash (MZ1, MK+1, PL1) are characterized by a strength increase of 36%, 29%, and 33%, respectively. On the other hand, samples with a 20% addition of compacted microsilica, Mikrosill+ microsilica, and fly ash (MZ2, MK+2, PL2) are characterized by a strength increase of 18%, 7%, and 5%, respectively. The results of the remaining samples are similar and amount to approximately 92% for the 10% content of additives and 79% for the 20% content of mineral additives. In the case of compressive strength, it can also be stated at the same time that the compressive strength does not depend directly on the specific surface area of the mineral additive used. Based on the compressive strength results, similarly to the bending strength, an analysis of the strength increase during the maturation of the samples was made for each additive, depending on its content. Figure 9 presents a summary of the obtained results of compressive strength after 7, 28, and 56 days of curing for all additives divided into 10% and 20% content. Figure 10 presents, similarly to the case of bending strength, the increases in compressive strength. For each sample, the reference value was the compressive strength after 7 days of curing. The increases in strength were presented as an increase in strength after 28 days and after 56 days relative to the strength after 7 days for each additive separately.

Based on the presented results, it should be stated that the applied mineral additives had a positive effect on the increase in compressive strength over time. In the case of using a 10% additive instead of cement, similar strength increases were obtained by samples with the addition of limestone flour, glass flour, basalt dust, and glass granulate. The second group of additives giving a similar effect on the increase in bending strength are compacted microsilica, microsilica microsill+, and fly ash. Fly ash is characterized by the greatest increase in compressive strength. Analyzing the results of using a 20% additive instead of cement, it can be stated that all additives have a similar effect on the increase in compressive strength; however, most of them, except for fly ash and microsilica microsill+, are characterized by a smaller increase in strength than the mortar samples without additives. In most cases, the increase in strength can be considered linear.

### 3.5. Dependence of Compressive Strength on Mineral Additive Parameters

**Figure 11:**
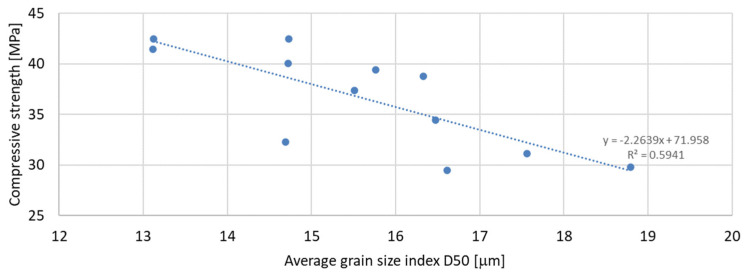
Dependence of compressive strength after 7 days on D50 index.

**Figure 12:**
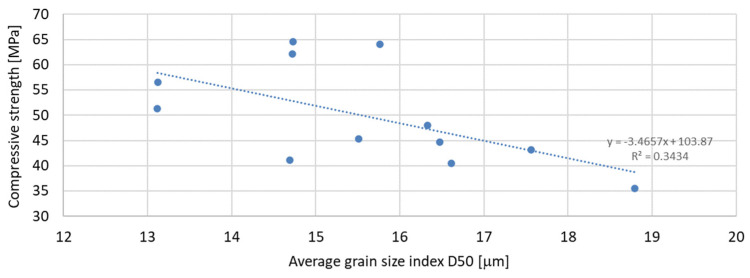
Dependence of compressive strength after 28 days on D50 index.

**Figure 13:**
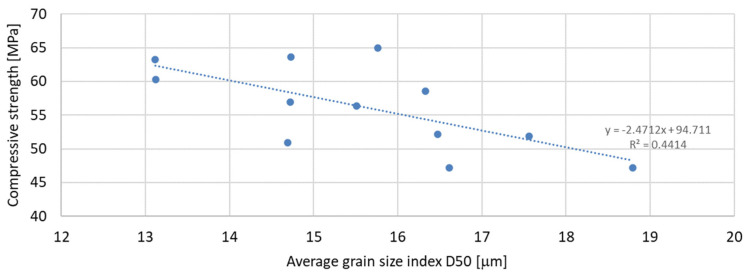
Dependence of compressive strength after 56 days on D50 index.

**Figure 14:**
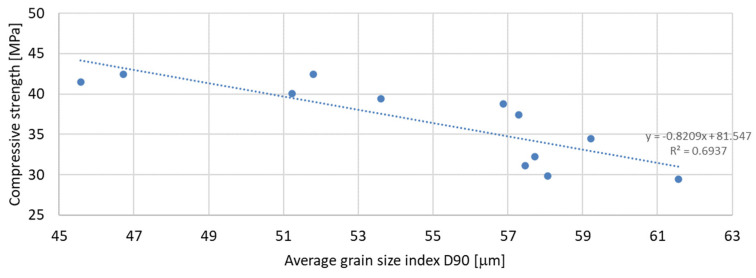
Dependence of compressive strength after 7 days on D90 index.

**Figure 15:**
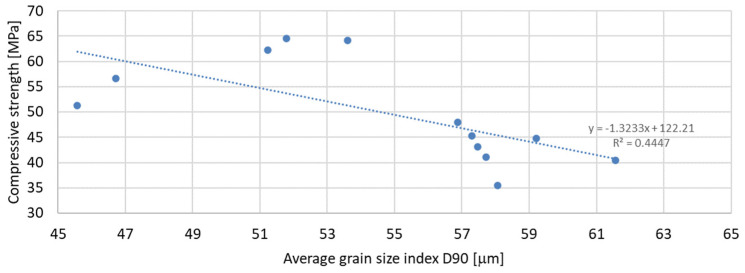
Dependence of compressive strength after 28 days on D90 index.

**Figure 16:**
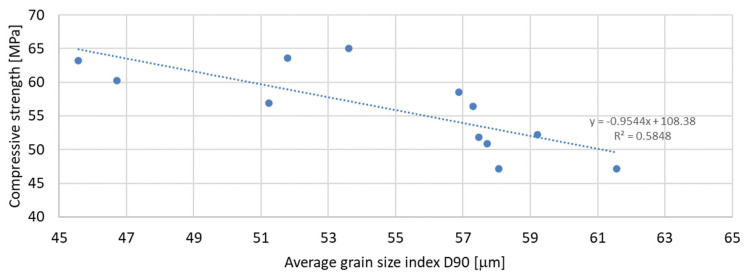
Dependence of compressive strength after 56 days on D90 index.

Based on the analysis of the obtained compressive strength results after 7, 28, and 56 days, an analysis of the dependence of compressive strength on the parameters of mineral additives such as the D50 index, D90 index, specific surface area, and SiO_2_ content was carried out. The percentage content of the additive in the mortar was taken into account in the tests of the dependence on compressive strength. Since the additive in the form of glass granulate is characterized by a D50 index above 334 μm and a D90 index above 521 μm, i.e., it can be classified to a greater extent as sand than as a fine-grained mineral additive, it was not included in the analysis of the effect on compressive strength. Figure 11, Figure 12 and Figure 13 present the dependence of compressive strength on the D50 index. On the other hand, Figure 14, Figure 15 and Figure 16 show the dependence of compressive strength on the D90 index.

Analyzing the above results, it can be stated that the highest dependence of compressive strength on the D50 index is characteristic of samples after 7 days of conditioning, for which the R-squared index is equal to 0.5941. Next in terms of convergence are the results of samples after 56 days of curing, for which the R-squared index is equal to 0.4414, while the lowest dependence on the D50 index is characteristic of samples after 28 days of curing, for which the R-squared index is equal to 0.3434. It should therefore be stated that compressive strength does not depend directly on the value of the D50 index.

**Figure 17:**
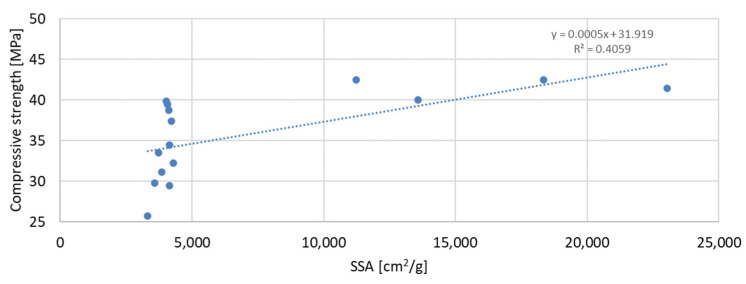
Dependence of compressive strength after 7 days on specific surface.

**Figure 18:**
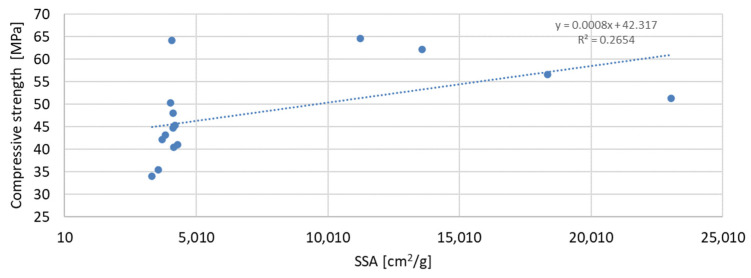
Dependence of compressive strength after 28 days on specific surface.

**Figure 19:**
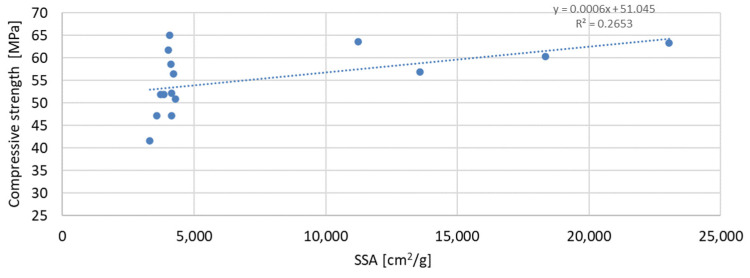
Dependence of compressive strength after 56 days on specific surface.

**Figure 20:**
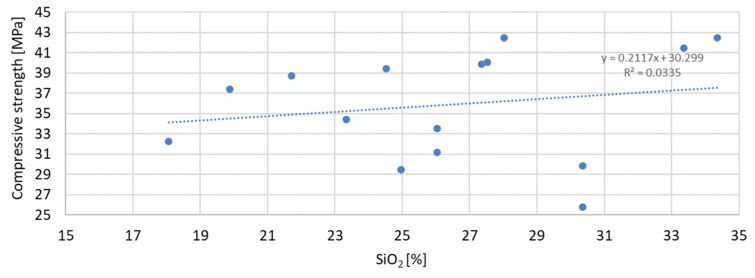
Dependence of compressive strength after seven days on SiO_2_.

**Figure 21:**
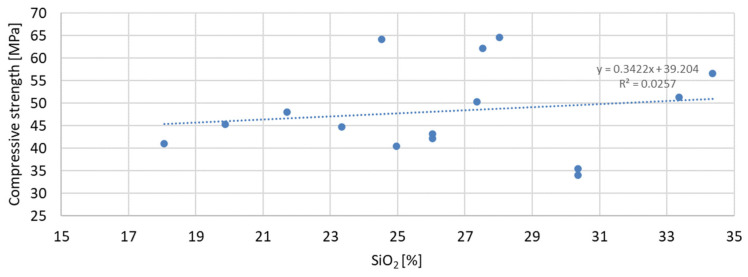
Dependence of compressive strength after 28 days on SiO_2_.

**Figure 22:**
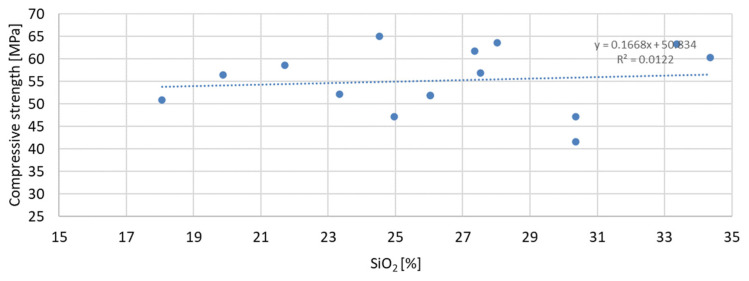
Dependence of compressive strength after 56 days on SiO_2_.

The above-presented dependencies show that the compressive strength depends much more on the D90 index than on the D50 index. For the seven-day strength, the R-squared index is the highest and equal to 0.6937; next in value is the R-squared index for the strength after 56 days of curing, equal to 0.5848; and the lowest, as in the previous case, is the strength after 28 days of curing, which is equal to 0.4447. Despite much greater dependencies, there is no significant effect of the grain size of the mineral additive on the compressive strength of the mortar samples observed in the tests. A much smaller correlation was obtained for the dependence of the compressive strength on the specific surface area of the mineral additives. In this analysis, the mineral additive in the form of glass grit was not omitted. The results of the obtained dependencies are presented in Figure 17, Figure 18 and Figure 19:

The greatest convergence of results is characteristic of samples after seven days of conditioning, for which the R-squared index of dependence on the specific surface area is 0.4059. Samples after 28 and 56 days of curing are characterized by the same dependence of results on the specific surface area. The R-squared index for them is 0.2654 and 0.2653, respectively. Therefore, in the case of the specific surface area, it should also be stated that compressive strength is not directly dependent on it. The lowest correlation of results was obtained for the dependence of compressive strength on the SiO_2_ content in the mortar mixture. The results of the obtained dependencies are presented in Figure 20, Figure 21 and Figure 22.

The presented correlation analyses of the dependence of compressive strength on SiO_2_ content in the mortar show that the highest correlation was obtained for samples after 7 days of curing and the lowest for samples after 56 days of curing, for which the R-squared indices are 0.0335 and 0.0122, respectively. The correlation results obtained from the analysis of the dependence of compressive strength on SiO_2_ content are over twenty times lower than in the case of the D90 index. It can therefore be stated that the SiO_2_ content in the applied mineral additives has the least effect on compressive strength.

### 3.6. Dependence of Flexural Strength on Mineral Additive Parameters

Since the parameters of mineral additives have a much smaller effect on the flexural strength than on the compressive strength, it was decided to compare only the results of the R-squared trend line index for the performed dependence analyses. The above results are presented in Table 6.

Based on the results presented in the table, it should be stated that the greatest correlation is characterized by the dependence of the flexural strength after 28 days of curing on the D90 index, which is 0.5958. For the D50 index, the above correlation is much smaller and amounts to only 0.2707. The smallest correlation is characterized by the dependence of the flexural strength after 7 days of curing on the SiO_2_ content and the flexural strength after 56 days of curing on the D50 index, which are 0.0006 and 0.0008, respectively. In the case of the compressive strength, the R-squared index for both correlations was 0.0335 and 0.4414, respectively. Therefore, it cannot be unequivocally stated that flexural strength depends on the analyzed indices.

## 4. Discussion

Analyzing the results of the tests carried out in terms of specific surface area and grain size, it can be clearly stated that the 7 mineral additives used have significantly different specific surface areas. The largest specific surface area in relation to cement is possessed by microsilicas, which are 18 and 23 times larger than cement. Limestone flour and basalt dust have a specific surface area similar to cement, and fly ash is slightly smaller. Glass flour is characterized by a specific surface area three times smaller than cement, and glass granulate is 32 times smaller. The initial thesis put forward in the tests that the specific surface area depends on the size of the material particles was not unequivocally confirmed in the tests because it was confirmed only in the case of microsilicas, glass flour, and glass granulate. According to the authors, the size of the specific surface area, in addition to the size of the grain, is influenced by its shape. The more angular the grain is, e.g., as a result of crushing, the greater its specific surface area. Analyzing the results of determining the consistency of the mortars, it can be concluded that the addition of 10 and 20% of microsilica and fly ash significantly reduced the consistency of the mortar. On the other hand, maintaining consistency was achieved with the addition of limestone flour, basalt dust, and glass flour. Attention should be paid to the results of testing the consistency of glass granulate (GS), which, despite the smallest specific surface area and the largest grains, caused a decrease in consistency in relation to the standard mortar without additives. Based on the determination of the flexural strength of mortars with 10% mineral additives, a clear increase in flexural strength over time should be noted. In the flexural strength test after 28 days of maturation, samples with the addition of 10% and 20% of microsilica (MZ1, MK+1, MZ2, MK+2) and with 10% fly ash (PL1) achieved greater flexural strength than samples without mineral additives. Based on the obtained results, it can be stated that the use of microsilica and fly ash additives significantly increases compressive strength. Samples with a 10% addition of compacted microsilica, Mikrosill+ microsilica, and fly ash (MZ1, MK+1, PL1) are characterized by higher strength by 36%, 29%, and 33%, respectively. The results of compressive strength obtained in the tests conducted for fly ash are different from the literature results. In the results presented in [38], no increase in compressive strength was obtained for the addition of 10% fly ash after both 7 and 28 days of maturation. In the case of 20% fly ash, a 12% decrease in strength was obtained after 7 days and a 15% decrease in compressive strength after 28 days of maturation of the samples. However, a significant increase in compressive strength was observed after 91 days for 10% fly ash. Also in the case of microsilicas in [39], decreases in strength were obtained in relation to the standard samples after 7 and 28 days of maturation, amounting to 19% and 14% for 10% microsilica and 32% and 35% for 20% microsilica, respectively. The differences obtained may result from a slightly different initial composition of the standard mortar. In the above tests, 500 g of cement and 170 mL of water were used, while in the tests presented in this article, 450 g of cement and 225 g of water were used, which has a significant impact on the consistency of the mortar at the compaction stage. Significant decreases in compressive strength were noted in the case of glass granulate (GS), in which after 7, 28, and 56 days, 33, 42, and 51 MPa were obtained, respectively. The results of the remaining samples are similar and amount to approximately 92% for 10% additive content and 79% for 20% mineral additive content. In the literature results, one can also observe the unfavorable influence of additives, e.g., glass flour, which added in the amount of 10% causes a decrease in compressive strength after 7 days by about 20% and after 28 days by about 16% [40]. On the other hand, the addition of lime flour in the amount of 10% causes a decrease in strength after 7 and 28 days of maturing by about 8% in relation to the standard mortar [41].

## 5. Conclusions

Based on the tests carried out involving the introduction of mineral additives to the standard mortar in the amounts of 10% and 20% as a substitute for cement, the following conclusions can be drawn:

1. Five types of additives: compacted microsilica, MIKROSILL+ microsilica, limestone flour, glass granulate, and fly ash, increased compressive strength after 7 and 28 days of maturation.
2. The highest increase in compressive strength was obtained for Microsilica 42% and Microsilica MIKROSILL+ 37%.
3. An increase in the flexural strength of the standard mortar was obtained for the additive MIKROSILL+ microsilica; for the remaining additives, the results obtained were comparable to or lower than the reference mortar.
4. The highest increase in flexural strength was obtained for Microsilica 9.9% and Microsilica MIKROSILL+ 7.5%.
5. The use of mineral additives in the form of compacted microsilica or MIKROSILL+ microsilica significantly increases the compressive and flexural strength and can therefore be used for the rapid reconstruction of military infrastructure.

## Figures and Tables

**Table 1:** Properties of CEM I 42.5 R cement.

Property	Unit	Value
Specific surface area	cm^2^/g	4124
Start of setting time	min	184
End of setting time	min	242
Change in volume	mm	1.0
Compressive strength		
After 2 days	MPa	30.1
After 28 days	MPa	60.2
Contents SO_3_	%	2.95
Contents Cl	%	0.089
Insoluble residue	%	0.57
Loss of ignition	%	3.33

**Table 2:** Chemical composition of the additives used.

Component	Unit	MZ	MK+	MW	MS	PB	GS	PL
SiO_2_	%	>80.0	>85.0	3.5	>65.0	38.2	>65.0	57.0
CaO	%	<3.5	<1.0		>8.0	15.2	>8.0	10.0
CaCO_3_	%			93.0				
FeO_3_	%			0.3	<0.2	15.9	<0.2	16.0
MgO	%			0.7	<0.4	7.7	<0.4	
SO_3_	%	<4.0	<2.0			0.2		
Na_2_O	%	<8.0	<0.5		>14.0	2.9	>14.0	
Al_2_O_3_	%				2.0	12.7	2.0	28.0
Cl^−^	%	<1.8	<0.3			0.07		

**Table 3:** Symbols of research samples.

Mineral Supplement	Contents of the Supplement 10%	Contents of the Supplement 20%
Compacted microsilica	MZ1	MZ2
Microsilica Mikrosill+	MK+1	MK+2
Limestone flour	MW1	MW2
Glass flour	MS1	MS2
Basalt dust	PB1	PB2
Glass granulate	GS1	GS2
Fly ash	PL1	PL2

**Table 4:** Results of the grain size test of mineral additives.

Addition	Indicator D50 [μm]	Indicator D90 [μm]	Specific Surface Area [cm^2^/g]
MZ	0.31	6.12	75,224
MK+	0.26	0.39	98,677
MW	8.13	61.11	4980
MS	28.64	62.82	1383
PB	17.75	80.33	4277
GS	334.61	521.54	131
PL	10.63	24.16	3610
CEM I	16.32	56.91	4192

**Table 5:** Flexural strength results.

Mineral Supplement	Flexural Strength after 7 Days/Standard Deviation [MPa]	Flexural Strength after 28 Days/Standard Deviation [MPa]	Flexural Strength after 56 Days/Standard Deviation [MPa]
P0	7.97/0.15	9.02/0.38	9.38/0.18
MZ1	7.27/0.29	9.14/0.10	10.31/0.43
MZ2	6.56/0.15	9.84/0.10	9.14/0.31
MK+1	7.62/0.20	10.2/0.33	10.08/0.16
MK+2	6.80/0.44	9.38/0.12	9.38/0.14
MW1	5.27/0.59	7.27/0.84	8.44/0.49
MW2	6.68/0.69	7.73/0.43	8.44/0.30
MS1	5.39/0.40	7.62/0.73	9.61/0.25
MS2	5.86/0.19	8.32/0.62	9.38/0.12
PB1	6.09/0.48	8.67/0.56	8.44/0.23
PB2	5.04/0.19	7.27/0.41	9.14/0.40
GS1	6.09/0.23	8.20/0.36	9.14/0.49
GS2	5.16/0.42	6.56/0.59	7.97/0.29
PL1	7.27/0.16	9.38/0.30	10.31/0.56
PL2	5.63/0.24	8.91/0.57	9.61/0.05

**Table 6:** R-squared index results for flexural strength dependence.

Analyzed Dependency	Flexural Strength after 7 Days [MPa]	Flexural Strength after 28 Days [MPa]	Flexural Strength after 56 Days [MPa]
Indicator D50	0.1716	0.2707	0.0008
Indicator D90	0.2547	0.5958	0.1573
Specific surface area	0.1636	0.3976	0.0747
Contents SiO_2_	0.0006	0.1236	0.0536

## Data Availability

No new data were created or analyzed in this study. Data sharing is not applicable to this article.
